# A Rice *Stowaway* MITE for Gene Transfer in Yeast

**DOI:** 10.1371/journal.pone.0064135

**Published:** 2013-05-21

**Authors:** Isam Fattash, Priyanka Bhardwaj, Caleb Hui, Guojun Yang

**Affiliations:** Department of Biology, University of Toronto Mississauga, Mississauga, Canada; Oregon State University, United States of America

## Abstract

Miniature inverted repeat transposable elements (MITEs) lack protein coding capacity and often share very limited sequence similarity with potential autonomous elements. Their capability of efficient transposition and dramatic amplification led to the proposition that MITEs are an untapped rich source of materials for transposable element (TE) based genetic tools. To test the concept of using MITE sequence in gene transfer, a rice *Stowaway* MITE previously shown to excise efficiently in yeast was engineered to carry cargo genes (*neo* and *gfp*) for delivery into the budding yeast genome. Efficient excision of the cargo gene cassettes was observed even though the excision frequency generally decreases with the increase of the cargo sizes. Excised elements insert into new genomic loci efficiently, with about 65% of the obtained insertion sites located in genes. Elements at the primary insertion sites can be remobilized, frequently resulting in copy number increase of the element. Surprisingly, the orientation of a cargo gene (*neo*) on a construct bearing dual reporter genes (*gfp* and *neo*) was found to have a dramatic effect on transposition frequency. These results demonstrated the concept that MITE sequences can be useful in engineering genetic tools to deliver cargo genes into eukaryotic genomes.

## Introduction

Eukaryotic genomes are rich in repetitive sequences, particularly transposable elements (TEs) that are capable of jumping from one genomic locus to another. Nevertheless, only a small number of TEs produce proteins called transposases to carry out transposition reactions. The majority of TEs do not encode functional transposases, but instead depend on transposases produced by autonomous elements for mobilization. One type of nonautonomous element is wrecked autonomous element, bearing mutations in the transposase coding sequences. In extreme cases, the entire transposase coding sequences are deleted [1], [2]. Another type of nonautonomous elements does not bear protein coding regions, often they share very limited (if any) sequence similarities to transposase coding elements in the genome. Despite the apparent absence of cognate autonomous elements for these orphan elements, many of them appear to be highly efficient in transposition, and therefore exist in high copy numbers in a genome. For example, the human SINE element *Alu* is mobilized by the transposase produced by L1 elements that share very little sequence similarity with *Alu* [3]. Over a million copies have accumulated in the human genome [4]. While the high copy number of *Alu* and other class I TEs is mainly attributed to the “copy and paste” transposition mechanism, dramatic amplification has also been observed for class II TEs that use a “cut and paste” transposition mechanism, which does not inherently favor copy number increase [5], [6]. The most abundant orphan type of non-autonomous class II elements are miniature inverted transposable elements (MITEs) [7].

MITEs are typically very short (<500 bp) and lack any protein coding sequences [8], [9], [10], [11], [12], but they appear to be very effective in transposition and can exist in high copy numbers. A MITE family usually has high intra-family sequence similarity, some even contain a large number of identical copies, suggesting their recent or current transposition activity [1], [10], [13]. However, among the hundreds of identified MITE families, few have apparent cognate autonomous elements [1], [2]. Therefore, most MITE families may have achieved amplification using transposases produced by non-cognate autonomous elements. During evolution, these MITEs may have evolved sequence motifs or structures that allow efficient transposition [7], [14]. Motifs and structures enhancing transposition are sought after features for genetic materials used in TE based genetic tools to deliver cargo genes during transgenesis and mutagenesis.

Canonical use of class II TEs for genetic vectors is to flank cargo genes with the terminal and subterminal sequences of the autonomous elements [15], [16], [17] [18], [19], [20], [21], [22], [23]. However, there are only a limited number of active autonomous element sequences to use. In addition, the activity of autonomous elements may be negatively regulated by the motifs on the subterminal sequences [14]. Orphan type non-autonomous elements with efficient transposition activity such as MITEs may provide additional or alternative sources for efficient TE-based genetic vectors.

MITEs are abundant and often found in or near genes, therefore they can be used as genetic markers. The maize MITE *Heartbreaker* (*Hbr*) was used to develop molecular markers for maize [24]. A total of 213 polymorphic fragments were mapped in 100 recombinant inbred lines derived from a cross between the maize inbreds B73×Mo17. *Hbr* markers are distributed evenly across the 10 maize chromosomes in the studied population. MITE markers (*Barfly*, *Pangrangja* and *Stowaway*) were also integrated into the genetic map of barley [25]. The 214 MITE based AFLP markers were shown to be superior over conventional AFLP mapping. The entire barley genome was covered with a limited set of MITE-based primers, consistent with that of an earlier study using MITEs for genetic mapping of barley genomes [26]. For rice, a *Stowaway* MITE *Pangrangja* and a *Tourist* MITE *Ditto* were used to reveal polymorphism between the parental lines of intraspecific F15 recombinant inbred lines, locating 78 markers of *Ditto* and 22 markers of *Pangrangja* [27]. In addition, a MITE in peanut was used to develop a DNA marker to differentiate chemically induced high-oleate mutants from the normal oleate peanut variety or naturally occurring high-oleate mutants [28]. Recently, the utility of MITEs was demonstrated in the analysis of wheat biodiversity and evolution[29], [30]. MITEs can also be used for gene tagging. The *mimp*1 element in the *Fusarium graminearum* genome was used to identify novel pathogenicity genes [31]. The *mPing* element is active in rice plants [1], [32], [33], [34]. Using transposase sources from *Ping* and *Pong*, the transposition activity of *mPing* has been demonstrated in transgenic heterologous host such as *Arabidopsis thaliana* [35]. Furthermore, *mPing* was recently shown to be useful in gene tagging of the soybean genome [36]. Considering the large repository of different MITE families in eukaryotic genomes and their capability of efficient transposition, MITEs are potentially rich sources of genetic materials to engineer genetic tools for transgenesis of both germinal and somatic cells. Despite the recent demonstration of transposition activities for a few other MITE elements including *MiHsmar*1 in human cell culture, *mimp*1 in fungus and *dTstu*1 in potato [2], [31], [37], the potential use of these MITEs for gene transfer remains unclear.

The most abundant type of MITEs in the rice genome is *Stowaway*. There are 25 families of rice *Stowaway*s with a total copy number of ∼20,000 [38]. These elements bear very short terminal inverted repeats (TIRs) and, like *Tc1/mariner* elements, they insert specifically at the dinucleotide “TA”. These *Stowaway*s are typical orphan TE families that do not have cognate autonomous elements in the genome. Using yeast as a heterologous host, it has been demonstrated that *Stowaway*s can be mobilized by transposases from rice *mariner*-like elements *Osmar*s [14], [39]. Particularly, one *Stowaway* element (Ost35) is excised by the *Osmar*14 transposase efficiently even though the same transposase excises a non-autonomous *Osmar*14 poorly. Paradoxically, the *Osmar*14 terminal sequences showed a stronger affinity than those of Ost35 to the transposase. Further analyses revealed a repressive motif present in the subterminal sequence of *Osmar*14 and, strikingly, the internal sequence of Ost35 was found to enhance transposition significantly. During these studies, an engineered Ost35 (named 14T32) with its TIRs replaced by those of *Osmar*14 showed a 20-fold increase in excision frequency. A mutated version of this hybrid element named T7 increased activity by an additional 3-fold [14]. This *Stowaway*-based hyper active element has the potential to be used as a vector to carry cargo genes for genetic manipulation. However, due to their natural existence in minimal sizes, *Stowaway* sequences may require their native (uninterrupted) forms to function in transposition. To explore the possibility of using MITE sequences as genetic tools to carry cargo genes, we tested the capacity of T7 to carry cargo genes and analyzed the transposition characteristics of T7-derived vectors.

Here we report the engineering of *Stowaway*-based constructs and the use of these vectors to carry cargo genes for delivery into the yeast genome. The cargo gene cassettes can be efficiently excised and inserted into yeast genomic DNA, introducing reporter genes into the yeast genome and generating mutations in yeast genes. The inserted elements can be remobilized and increase copy number in a genome. Interestingly, the orientation of a cargo gene (*neo*) was found to have a dramatic influence on the transposition efficiency of the cassette.

## Materials and Methods

### Strains and constructs

Yeast strain DG2523 (*MATalpha ura3-167 trp1-hisG leu2-hisG his3-del200 ade2-hisG*) was used in the study. The aminoglycoside 3′ phosphotransferase gene (*neo*) from Tn903 (Tn601) was amplified from pITY3 [40], [41] with 5′ phosphorylated primers 5′ ggatccgggttacattgcacaaga 3′ and 5′ ggatccggcgccgtcccgtcaagt 3′. Gel purified PCR product was inserted at the *Bsa*AI site on the plasmid pT7, resulting in pT7-*neo*. The green fluorescence protein gene (*gfp*) coding sequence was amplified from pBin-mGFP5-er [42] using *Pfu* DNA polymerase (iProof, Biorad) with primers 5′ atctcaggatccatgagtaaaggagaagaacttttcactggaga 3′ and 5′ atctcagaattcttaaagctcatcatgtttgtatagttcatccatgc 3′, the amplicon was cloned between *Bam*HI and *Eco*RI sites on the backbone of pOsm14Tp so that *gfp* is expressed from the *gal*1 promoter. The fragment containing the *gal*1 promoter and the *gfp* fragment and *cyc*1 terminator was amplified with *Pfu* DNA polymerase with primers 5′ ctagtacggattagaagccgccg 3′ and 5′ gtttaaacgcaaattaaagccttcgagcgtcc 3′ and cloned at the *Bsa*AI site of pT7, resulting in plasmid pT7-*gfp*. To construct pT7-*gfp-neo* and pT7-*gfp*-*oen*, the *Pme*I restriction site that was attached to the reverse primer to amplify the *gfp* gene was used to clone the *neo* gene. The orientation of the constructs was determined from the gel banding pattern after restriction enzyme digestion. The *neo* gene was cloned into the *Bsa*AI site of pOst35 to make pOst35-*neo*. To construct p14TIR-*neo*, PCR primers for *neo* gene amplification were attached with the terminal 32 bp sequences of *Osmar*14 on the 5′ end. PCR products using *pfu* were cloned into the *Hpa*I site inside the *ade*2 gene on pWL89A [43].

### Yeast excision assay

Yeast excision assays were performed according to the procedure described previously [14], [39]. Briefly, yeast competent cells were transformed with pOsm14Tp and the constructs described above and double transformants were selected on yeast dropout medium lacking histidine and uracil in the presence of 2% galactose and 1% raffinose to induce transposase production. After incubation at room temperature, cells from a colony were resuspended in 50 µl of water and 49 µl plated on medium lacking adenine to select for *ade*2 revertants. The reversion frequency was calculated as the number of revertants per cell per generation. The number of generations was estimated using the formula: N = log_2_T, where N is the number of generations and T is the number of total viable cells. The number of total viable cells of a colony was determined by plating 49 µl diluted (1.6×10^5^) cell suspension on YPD plates. Genomic DNA extracted from revertants was used in PCR to amplify the donor sites using primers 5′ gggttttccattcgtcttgaagtcgaggac 3′ and 5′ catttccacaccaaatataccacaaccggga 3′. PCR products were sequenced at The Center for Applied Genomics (The Hospital for Sick Children, Toronto) and the donor sites were inspected for transposition footprints.

### Yeast insertion assay

To detect insertion of cargo gene cassettes into the yeast genome, colonies incubated on transposase induction medium were cultured in YPD to allow some cells to lose the transformed plasmids bearing the original cargo gene cassettes. Then a appropriately diluted YPD culture suspension was plated onto complete synthetic medium (Sunrise Science Products, Inc., CA) lacking histidine but containing 1% 5-fluoroorotic acid (5-FOA, BioBasic) to obtain well separated colonies. This medium selects for cells that have lost the plasmid carrying the cargo gene construct, but retained the transposase construct (pOsm14Tp). For pT7-*neo*, the colonies on 5-FOA medium were replica plated onto YPD plates containing the antibiotic G418 (100 mg/L, BioShop) to select for cells containing a transposed copy of the *neo* gene. For constructs bearing the *gfp* gene, colonies grown on plates containing 5-FOA were examined under blue light to identify colonies with green fluorescence using a SteREO Discovery 12 microscope (Carl Zeiss). The insertion frequency was calculated as the number of G418 resistant colonies (for T7-*neo*) or GFP expressing colonies (for T7-*gfp*) divided by the total number of 5-FOA tolerant colonies. The presence or absence of the T7-*neo* cassette was verified by PCR amplification using primers 5′ tactccctccgtcccagaaagaagggattc 3′ and 5′ tgctcgatgagtttttctaatcagaattggttaattggttgt 3′. The presence or absence of the T7-*gfp* cassette was verified by PCR amplification using primers 5′ tactccctccgtcccagaaagaagggattc 3′ and 5′ atctcagaattcttaaagctcatcatg 3′. The presence or absence of the donor plasmid was verified by PCR amplification of the *ade*2 coding sequence using primers 5′ gggttttccattcgtcttgaagtcgaggac 3′ and 5′ catttccacaccaaatataccacaaccggga 3′ and by culturing the colonies in complete synthetic medium with or without uracil.

### Inverse PCR and insertion site sequencing

To determine the insertion sites of the transposed elements, yeast genomic DNA was extracted from liquid culture of individual colonies using E.Z.N.A.® Yeast DNA Kit (Omega BioTek). Genomic DNA (200 ng) was digested with *MseI* and ligated with T4 DNA ligase (NEB) in a 10 µl reaction at 16°C for 16 hours. The digestion/ligation product was diluted 100 times and 1 µl was used as a template for 20 µl PCR reaction using 2× PCR Master Mix (NEB) with primers 5′ ggtatttttgtcattctccatctcc 3′ and 5′ tgctgagggtaggtaggatggta 3′. Nested PCR was performed using 1 µl of the initial PCR product using 5′ tcacctcgggatgccaggaa 3′ and 5′ tgggacaaaattcaaactccaga 3′ primers. Individual gel bands from the amplified products were gel extracted and subsequently sequenced at The Center for Applied Genomics with the PCR primer 5′ tgggacaaaattcaaactccaga 3′. Genomic locations and annotations of insertion sequences were retrieved from *Saccharomyces* Genome Database (SGD) at www.yeastgenome.org.

### Genomic DNA blot analysis

Yeast genomic DNA (∼200 ng) was digested with *Nde*I and *Xho*I separated on an agarose gel (1%). DNA was electro-blotted onto nylon membrane using Mini Trans-Blot® Module (BioRad). Gel purified PCR product of T7-*neo* fragment amplified from pT7-*neo* was used as the template for probe preparation. Probe preparation and hybridization were performed using DIG high prime DNA labeling and detection starter kit I (Roche) according to the manual.

### Serial culture of yeast cells

Cultures of yeast bearing only a transposed copy of the T7-*neo* cassette were maintained continuously in the lab. Every 2–3 days, 200 µl culture was sub-cultured in 1.8 ml fresh complete synthetic liquid medium lacking histidine but containing 2% galactose and 1% raffinose at 30°C. An aliquot of 50 week old culture was plated onto solid medium lacking histidine and containing 2% galactose and 1% raffinose to obtain single colonies and to further induce transposition.

## Results

### 1. Excision of *Stowaway*-based cargo gene cassettes

**Figure 1:**
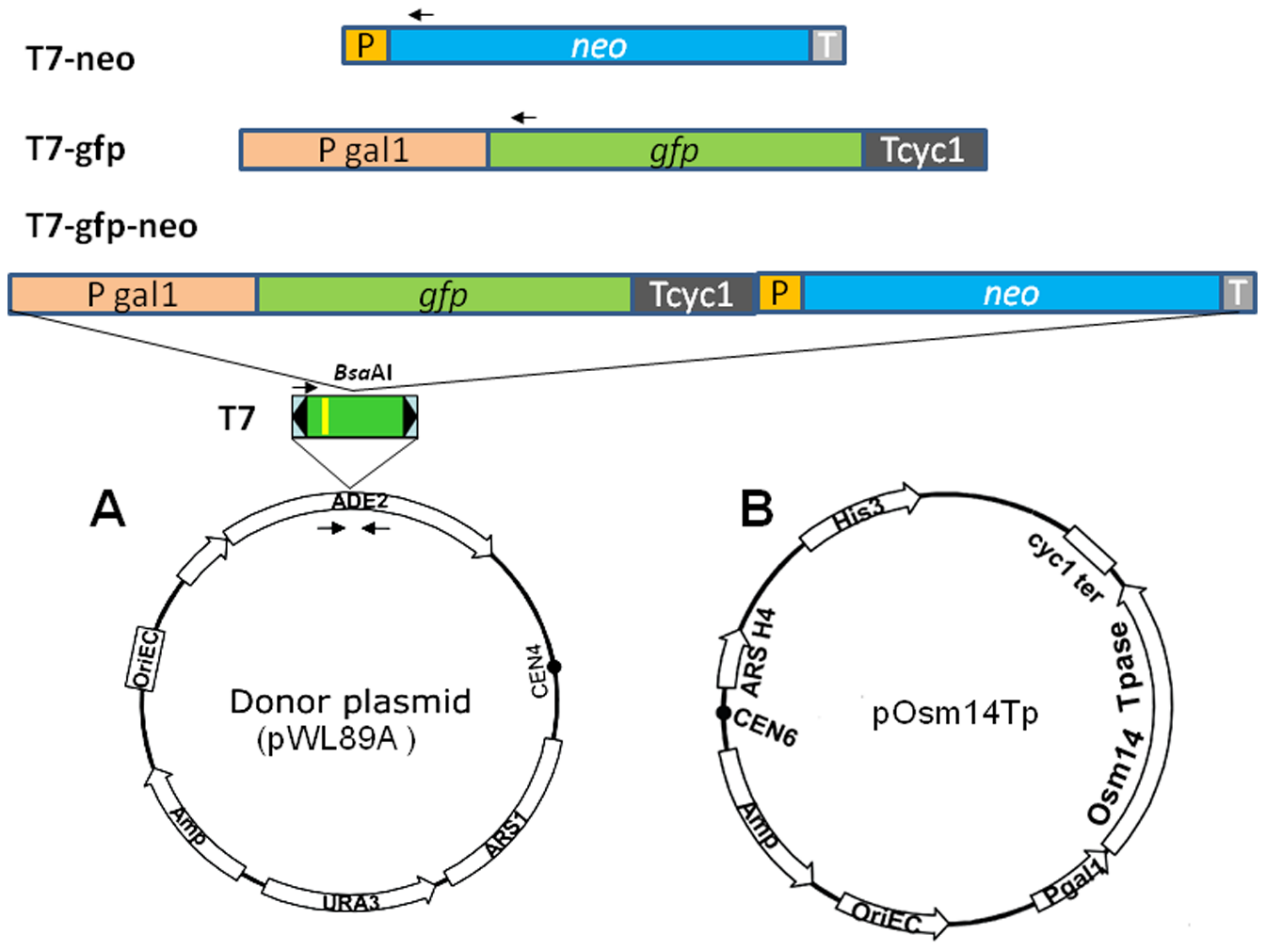
Schematic of hybrid *Stowaway* T7 based plasmids. Vertical yellow line, mutated site on T7; P, promoter for *neo* gene; T, terminator of *neo* gene; Pgal1, *gal1* promoter; T*cyc*1, terminator of *cyc1*. Donor plasmids are based on the pWL89A backbone. Constructs lengths are to scale. The plasmid pRS413 that does not carry the transposase gene was used as the control. No *ade*2 revertant was obtained on any control plate. Small arrowheads, positions for PCR primers to check the presence or absence of corresponding genes. The first three tracks show three different constructs used for integration at the *Bsa*AI site (as mentioned in the text).

**Figure 2:**
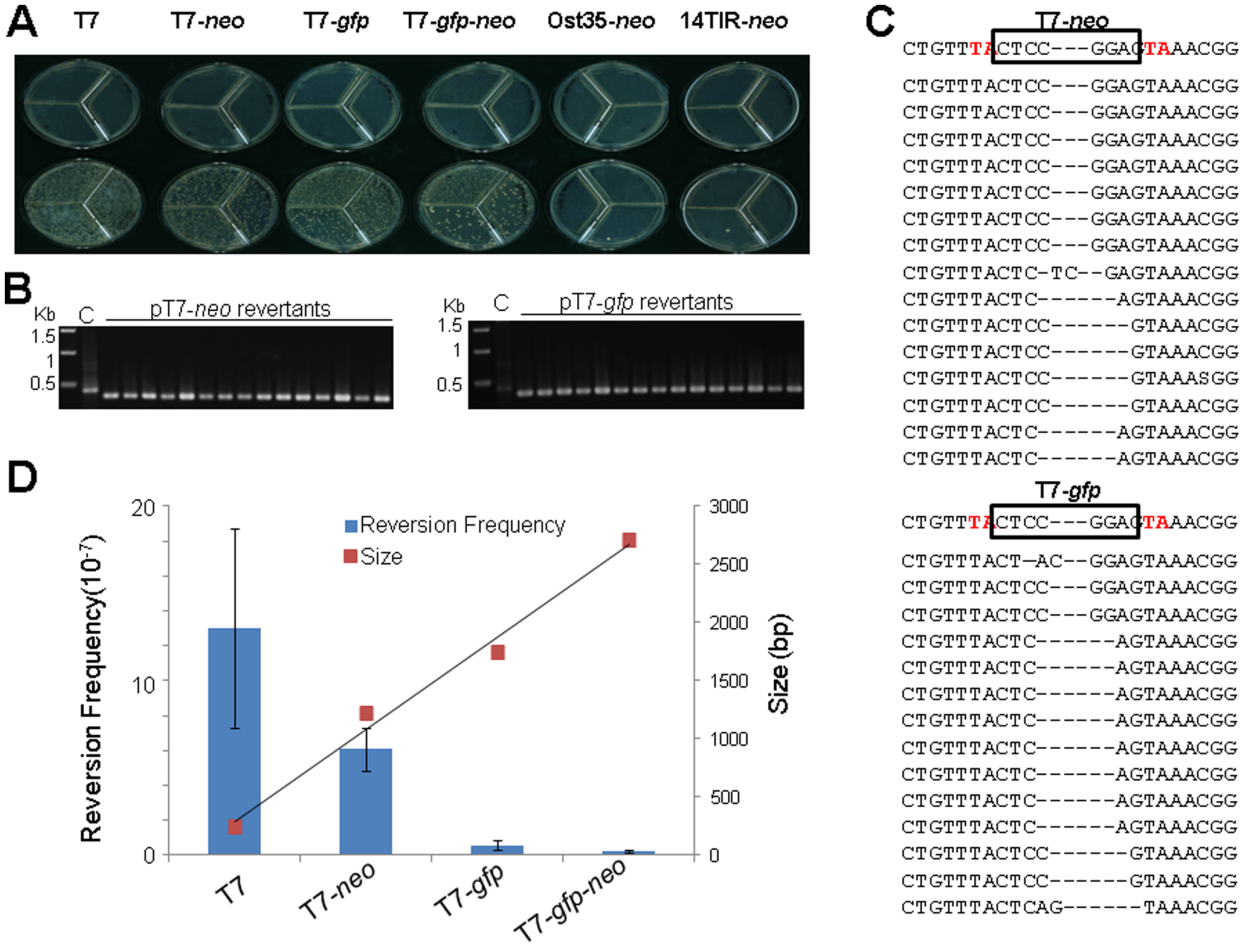
Excision assay of T7 derived constructs in yeast. (A) plates of medium lacking adenine in the presence (bottom row) and absence of transposase sources (top row), cells in each sector on a plate were from a separate colony incubated on medium lacking histidine and uracil for 14 days. (B) PCR amplification of the donor sites in the *ade2* revertants. C, plasmid control; (C) Sequences of the donor sites in the *ade2* revertants. Red letters, target site dinucleotide “TA”. Dashes, empty space. The donor sites containing TEs are shown on top of the donor sites containing footprints. (D) Reversion frequency for the constructs T7, T7-*neo*, T7-*gfp*, T7-*neo*-*gfp*. The reversion frequency (left y-axis) is plotted against the length of the cargo cassettes (right y-axis). Error bars, standard error of the mean for three independent replicates. Plates were incubated on transposase induction medium for nine days.

The hybrid *Stowaway* element T7 (240 bp) is hyper active in excision [14]. Cargo genes *neo* (1,213 bp), *gfp* (1,740 bp) and *gfp-neo* (2,713 bp) were cloned into the *Bsa*AI site in the middle of T7 (Fig. 1A). In addition, the *neo* gene was also cloned into the *Bsa*AI site of Ost35 (Ost35-*neo*) and between the TIR sequences of *Osmar*14 (14TIR-*neo*). After co-transformation with the plasmid (pOsm14Tp) bearing the *Osmar*14 transposase gene (Fig. 1B), colonies were grown on galactose medium to induce transposase required for the excision assay [14], [39]. As expected, no *ade*2 revertant was obtained from any of the control plasmid lacking the transposase gene. Hundreds of *ade*2 revertant colonies were obtained from the positive control pT7 and all vectors derived from pT7 (Fig. 2A, Table S1). Only one colony was obtained for Ost35-*neo* and 14TIR-*neo* (Fig. 2A). These results suggest that gene cassettes carried in T7 are excised efficiently.

Genomic DNA extracted from the revertants was used for PCR amplification of the donor site inside the *ade*2 coding sequence. Compared to the plasmid control, all of the revertants resulted in shorter PCR products with the size of about 300 bp, indicating the loss of the elements from the donor sites in the revertants (Fig. 2B). Sequencing of the PCR products confirmed the loss of the element and revealed footprints typically containing relic residues from the end of the TE left at the donor sites. Therefore, these revertants resulted from transposition events that have the excision sites repaired to restore the *ade*2 reading frame (Fig. 2C).

### 2. Cargo gene sizes and excision frequency

Among the constructs, pT7-*neo* has the highest reversion frequency (6.07×10^−7^ per cell per generation), about half of that for T7. The reversion frequency for T7-*gfp* (5.77×10^−8^ per cell per generation) is about one tenth of that for T7-*neo* (Fig. 2D, Table S2). The reversion frequency for T7-*gfp*-*neo* (2.17×10^−8^ per cell per generation) is about one thirtieth of that for T7-*neo* even though hundreds of *ade*2 revertants can be reproducibly obtained from a single colony bearing the construct. When the frequencies were plotted against the sizes of the elements (240, 1,213 bp, 1,740 bp and 2,713 bp), a negative correlation between the reversion frequencies and the sizes of the cassettes is apparent (Fig. 2D, Table S2). Similar observations have been reported for other TEs such as *Mos*1, *IS*50 and *IS*1 [44], [45], [46]. However, this negative correlation between cargo size and excision frequency can be disturbed by the configuration of cargo genes as described in “Effect of Orientation of *neo* gene on transposition efficiency”.

### 3. Insertion of cargo gene cassettes

**Figure 3:**
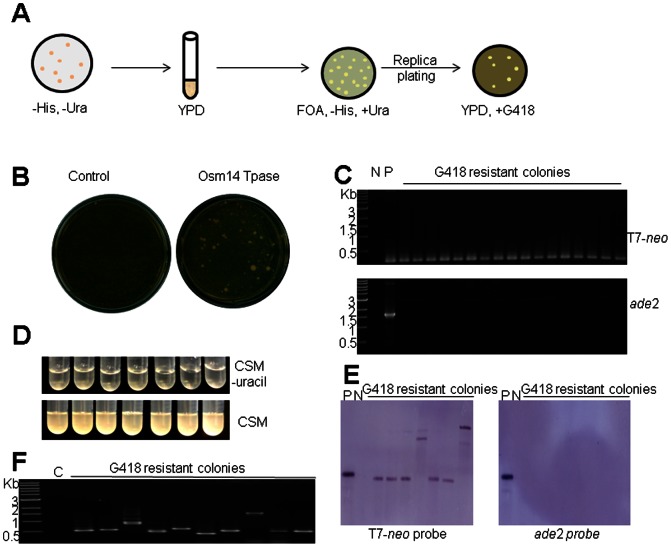
Insertion of T7-*neo*. (A) schematic experimental procedure to select for genomic insertion of T7-*neo* (see Materials and Methods); (B) selection of G418 resistant colonies in the presence (right) and absence of (left) transposase sources; (C) PCR amplification of *ade*2 gene coding sequence; N, negative control using DG2523 genomic DNA; P, positive control using plasmid pT7-*neo*. (D) growth of G418 resistant colonies on complete synthetic medium with (bottom) or without (top) uracil. (E) Genomic DNA blot analysis. P, plasmid (pT7-*neo*) control; N, negative control of yeast genomic DNA not containing T7-*neo* fragment. Both images were from the same blot but hybridized with different probes. (F) Inverse PCR amplification of insertion sites in G418 resistant colonies. C, control using DG2523 yeast genomic DNA. Plates were incubated on transposase induction medium for two weeks.

**Figure 4:**
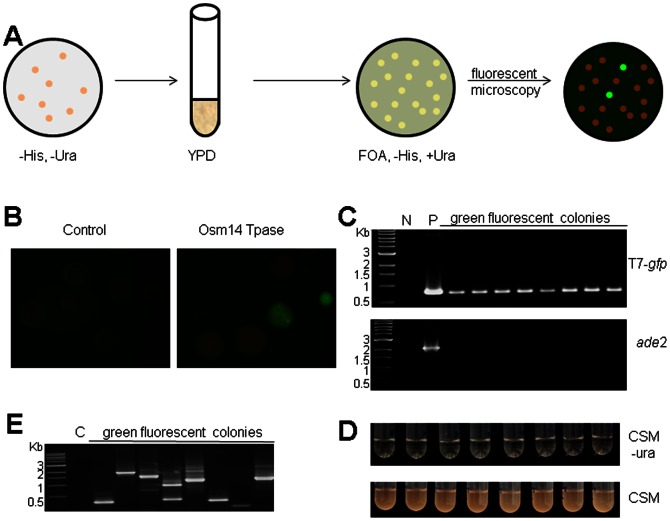
Insertion of T7-*gfp*. (A) schematic experimental procedure to identify genomic insertion of T7-*gfp* (see Materials and Methods); (B) Colonies in the presence (right) and absence of (left) transposase sources; (C) PCR amplification of *ade*2 gene coding sequence; N, negative control using DG2523 yeast genomic DNA; P, positive control using plasmid pT7-*gfp*. (D) growth of green fluorescent colonies in complete synthetic medium with (bottom) or without (top) uracil; (E) Inverse PCR amplification of insertion sites for green fluorescent colonies. C, control using DG2523 yeast genomic DNA. Plates were incubated on transposase induction medium for two weeks.

To detect insertions of the excised gene cassettes, yeast colonies carrying pOsm14Tp and a T7 based vector (pT7-*neo* or p T7-*gfp*) were plated on transposase induction medium. Cells in these colonies were allowed to lose their plasmids by subculturing in YPD medium without selection pressure. Cells that have lost the donor plasmid harboring the *ura*3 gene were counter selected on medium containing 5-FOA (Fig. 3A). Previous studies suggested that about 1% of the yeast cells carrying the CEN/ARS sequences will lose the plasmid every generation [47], [48]. For pT7-*neo*, colonies growing on 5-FOA were selected for the presence of *neo* gene by G418 resistance (Fig. 3B). The insertion frequency for T7-*neo* was determined to be 1.12±0.27×10^−3^ of the 5-FOA tolerant colonies (per cell per generation). Twenty-three colonies were picked for genomic DNA extraction and subsequent PCR amplification to check whether the cargo gene and the *ade*2 gene were present. All of the analyzed 23 colonies contained the cargo gene, but the *ade*2 gene was not amplified in any of them, confirming loss of the donor plasmid (Fig. 3C). In addition, these colonies were unable to grow in medium lacking uracil, indicating the loss of the donor plasmid (Fig. 3D). Seven genomic DNA samples were also used for DNA blot analysis. While none of the seven colonies contained the *ade*2 gene, they all contained the cargo genes. Based on the presence of different sized digestion fragments, some of the insertions are apparently at different genomic locations (Fig. 3E). A similar procedure was used to detect insertions of T7-*gfp* (Fig. 4A). Colonies growing on medium containing 5-FOA were examined for green fluorescence under blue light (Fig. 4B). The genomic insertion frequency for T7-*gfp* was determined to be 1.82±0.93×10^−4^ (per cell per generation). PCR amplification using yeast DNA was performed to determine the presence or absence of the *gfp* gene and the *ade*2 gene in yeast. While none of the 8 analyzed colonies carried the *ade*2 gene, all of them contained the T7-*gfp* cargo gene cassette (Fig. 4C). In addition, these colonies were unable to grow in medium lacking uracil (Fig. 4D). These results suggest that cargo genes transposed from the donor plasmids into yeast DNA.

To determine insertion site sequences, inverse PCR was performed and the PCR products were sequenced (Fig. 3F, 4E). All of the 25 obtained insertion sites are at the dinucleotides “TA” (Table S3), 16 (64%) are in genes and 9 (36%) are in intergenic regions. The haploidy of the strain used in this study may have lowered the percentage of insertions in coding sequences due to reduced fitness or lethality resulted from mutations of essential genes, nevertheless, the 64% insertion in genes is within the range of the ∼70% protein coding content of the genome. Interestingly, while all of the insertion sites from T7-*gfp* and T7-*gfp*-*oen*, a construct bearing the *neo* gene in opposite orientation to that in T7-*gfp*-*neo*, are on yeast chromosomes (Table S3), 7 (36.8%) from T7-*neo* are on the yeast 2 µ plasmid. Since the colonies bearing insertions for the first two constructs were identified by GFP expression, the selection on G418 for T7-*neo* may have arbitrarily enriched cells bearing insertions on the 2 µ plasmid. Since yeast strains often carry the 2 µ plasmid at 50–100copies/cell [49], the *gfp* marker appears to be better at generating insertions into chromosomes.

### 4. Remobilization and copy number increase

**Figure 5:**
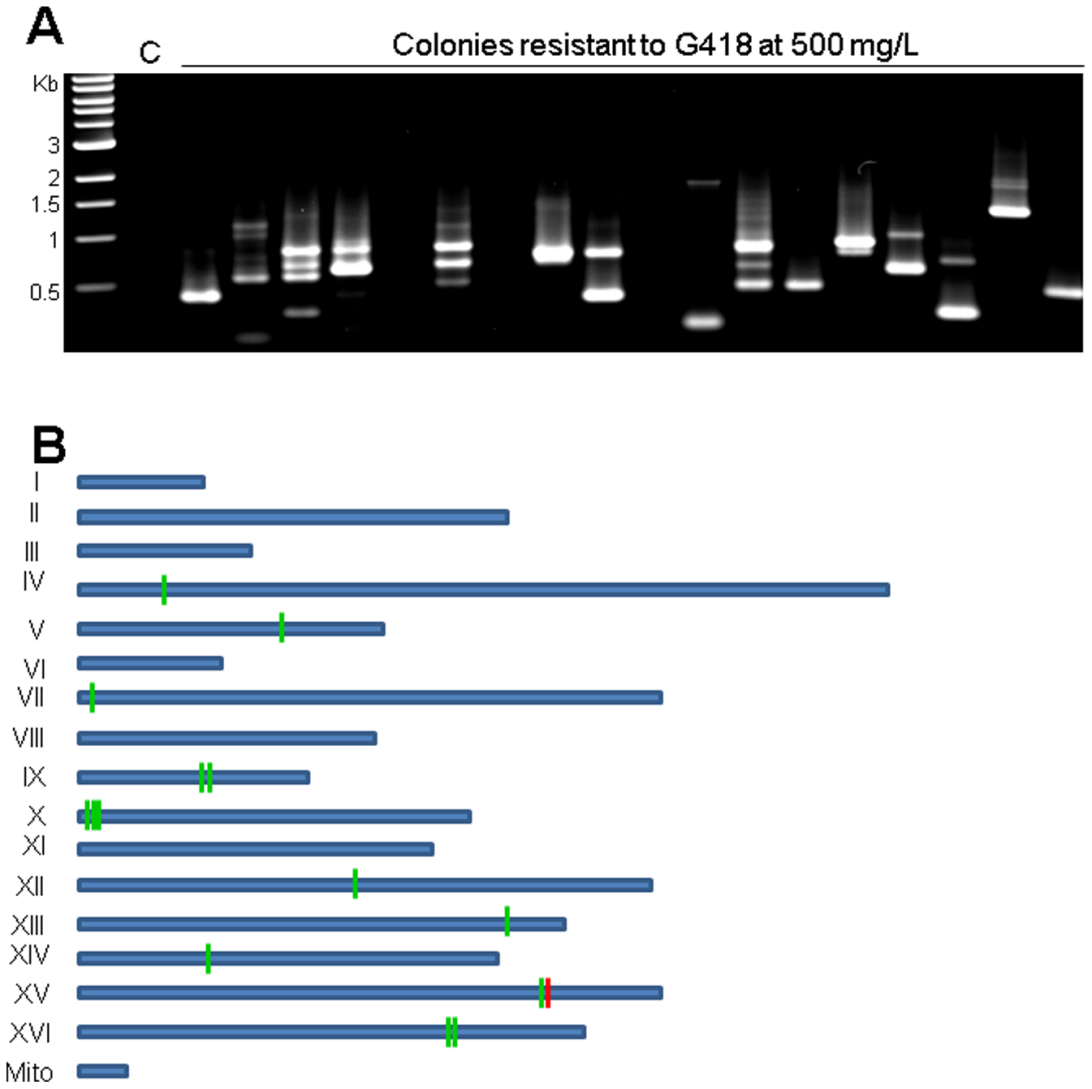
Remobilization and copy number increase. (A) Inverse PCR amplification of T7-*neo* insertion sites of the colonies on higher level of G418 (see Materials and Methods). C, negative control using the genomic DNA of the yeast strain. (B) T7-*neo* insertion sites in Table S4 mapped on yeast schematic chromosomes. Short vertical bars, insertion sites with the red bar representing the inferred first insertion on chromosomes.

Increase in copy number of a TE element depends on remobilization of an existing element. The *neo* gene was shown to have a dosage dependent resistance to G418, therefore a higher copy number of *neo* can be enriched on medium containing a higher concentration of G418 [40]. To understand whether a primary insertion of a T7-*neo* gene cassette can be remobilized and result in an increase in copy number, yeast strain L7 containing an initial insertion of T7-*neo* into the 2 µ plasmid was used. The yeast strain was serially sub-cultured in liquid synthetic medium containing galactose for transposase induction but lacking histidine to retain the transposase plasmid (see Materials and Methods). This condition favors the loss of the 2 µ plasmid from yeast cells [50]. An aliquot was sub-cultured on solid transposase inducing medium to obtain single colonies. Cells from a randomly picked colony were plated onto medium containing a relatively high level of G418 (500 mg/L) to select for cells expressing a relatively high level of *neo* expression. The insertion sites in these cells were determined by inverse PCR and subsequent sequencing of the PCR products (Fig. 5A). None of the obtained insertion sites are in the 2 micron plasmid, suggesting the loss of the 2 micron plasmid and remobilization of the original insertion into a yeast chromosome location (Table S4). Among the 20 analyzed colonies, 12 exhibited multiple bands in inverse PCR, and at least eight were confirmed to have two or more genomic copies of the gene cassette. Two contained at least three copies. While an early genomic insertion site in this lineage can be inferred to be at chromosome XV at 81,298 because of its high frequency of occurrence, the exact order of transposition events in this lineage is not clear. These results suggest that primary insertions of the gene cassette can be remobilized and result in an increase in the copy number of cargo genes.

### 5. Linked and unlinked transposition

**Figure 6:**
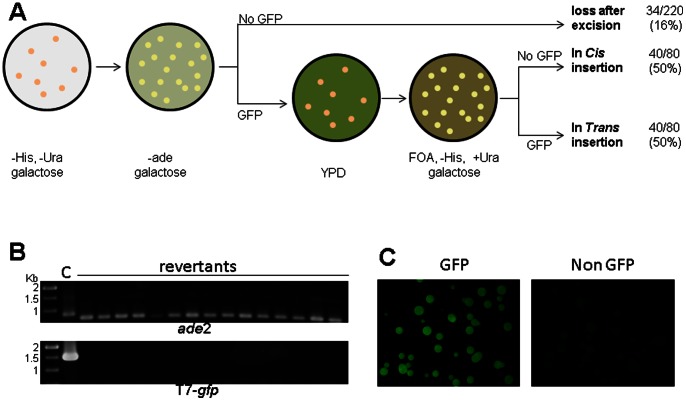
Linked and unlinked insertion rate of T7-*gfp*. (A) Schematic experimental procedure to determine the rate of loss of the excised elements, linked and unlinked insertion; (B) PCR amplification of the donor sites in *ade*2 coding sequences (top) and the cargo gene cassette T7-*gfp* in the *ade*2 revertant colonies that do not express GFP. C, pT7-*gfp* plasmid control; (C) Presence (left) or absence (right) of GFP expression in the colonies that have lost the donor plasmids (on medium containing 5-FOA).

*Tc1/mariner* elements have been shown to have a preference to transpose locally [51]. An excised element can be inserted into a linked DNA molecule (linked) or into an unlinked DNA location (unlinked). To investigate whether T7 derived cargo vectors have a preference to transpose to linked DNA molecules, we determined the ratio of linked and unlinked transposition for T7-*gfp* construct (Fig. 6A). In an excision assay using pT7-*gfp*, *ade2* revertants were selected on medium containing galactose to induce GFP expression. Among the revertants, 186 of 220 (∼84%) showed green fluorescence, suggesting that 16% of the revertants lost the *gfp* gene. The absence of T7-*gfp* in these colonies was confirmed by PCR amplification (Fig. 6B). The colonies expressing GFP were allowed to lose the donor plasmid by culturing in YPD medium. Cells missing the donor plasmid were selected on medium containing 5-FOA and galactose. Genomic insertions of the T7-*gfp* results in GFP expression in these colonies and donor plasmid insertions of T7-*gfp* gene results in the loss of GFP expression in these colonies. Therefore, either all of the colonies growing on this medium express GFP (unlinked insertion) or none of the colonies express GFP (linked insertion) (Fig. 6C). Among the 80 analyzed *ade*2 revertant colonies, 40 (50%) resulted in colonies expressing GFP on 5-FOA plates containing galactose, suggesting that 50% of the excised elements inserted linked into new loci on the donor plasmid and 50% inserted *unlinked* into yeast genomic DNA (Table S5). In addition, when the insertion sites of T7-*neo* listed in Table S4 were mapped onto the schematic yeast chromosomes (Fig. 5B), four clusters of doublet or triplet insertion sites were observed, probably resulted from local transposition events.

### 6. Effect of Orientation of *neo* gene on transposition efficiency

**Figure 7:**
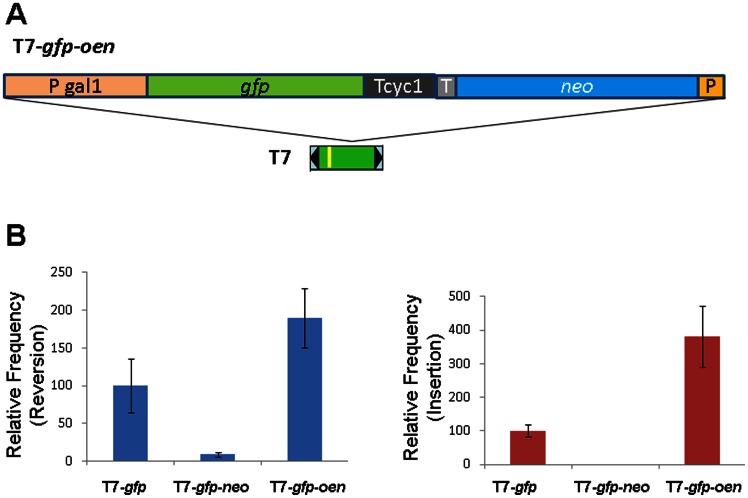
Effect of the orientation of *neo* gene on transposition efficiency. (A) schematic structure of the T7-*gfp*-*oen* cargo gene cassette. Legends are the same as those in Fig. 1. (B) Relative *ade*2 reversion efficiency (left) and cargo gene cassette insertion frequency (right) of the three constructs. The mean values of both reversion frequency and insertion frequency for T7-*gfp* were arbitrarily designated as 100%. Error bar, standard error of the mean for six independent replicates.

As described above, the *gfp* reporter resulted in a higher proportion of chromosomal integrations compared to the *neo* reporter (Table S3). The *neo* gene is useful for identifying remobilization and copy number increase. Therefore a dual gene cassette is a desirable construct to achieve these purposes. However, as shown in Fig. 2, the transposition frequency of a dual cassette in pT7-*gfp*-*neo* is significantly lower than that of the T7-*neo* and T7-*gfp*. We tested a construct bearing both *gfp* and *neo* but with the *neo* gene cloned in a different orientation from that on pT7-*gpf*-*neo*, therefore named as T7-*gfp*-*oen* (Fig. 7A). This construct was used to perform excision and insertion assays in comparison with T7-*gfp* and T7- *gfp*-*neo*. Strikingly, the *ade*2 reversion frequency for pT7-*gfp*-*oen* was about 20-fold of that for pT7-*gfp*-*neo* and about two-fold of that for pT7-*gfp*. Similarly, the insertion frequency for pT7- *gfp*-*oen* was nearly four-fold of that for pT7-*gfp* (Fig. 7B). Therefore, pT7-*gfp*-*oen* is an efficient vector both for the selection of insertions into genomic locations and for detecting copy number increase of the cargo gene construct. These results also suggest that, when large cargo cassettes are used, transposition efficiency may be enhanced with a specific cargo gene configuration that breaks the negative correlation between cargo size and transposition frequency observed for a number of elements [44], [45], [46].

## Discussion

In our studies, a *Stowaway* sequence was shown to be useful for gene delivery and mutagenesis in yeast. Even though a cargo gene cassette carried by the original MITE Ost35 did not result in efficient transposition activity, the internal sequence of this MITE is critical to the high transposition efficiencies for the hybrid *Stowaway* T7 based vectors. Even though this *Stowaway* has not been used for gene transfer in rice or other higher eukaryotes, the results obtained from the studies in yeast demonstrated the capability of MITE sequences in gene transfer. The fact that the rice MITE can be used to transfer genes into yeast suggests that they may also be useful for gene transfer in other heterologous hosts. The numerous MITE families in eukaryotic genomes can be a rich source of materials for TE based genetic vectors. However, the exact transposase sources for most of MITE families remain to be determined.

In this study, a surprising increase of transposition efficiency was observed simply by inverting the *neo* gene on the dual reporter gene vector. With few exceptions, conventional wisdom expects a negative correlation between the construct sizes and transposition efficiencies 44,45,46,52. This was observed for T7, T7-*neo*, and T7-*gfp-neo*. However, the plasmid containing the inverted *neo* (pT7-*gfp*-*oen*) did not follow the trend. The increased activity for pT7-*gfp*-*oen* is attributable to enhancement caused by the inverted orientation of *neo* gene. Interestingly, when the same *neo* gene was inserted into the *Mariner* element *Mos*1, no difference in transposition frequency was observed even when the *neo* gene was reversed in an *in vitro* assay [46]. One explanation for this observation can be due to the non-specific binding between the transposase and the *neo* sequence. Alternatively, changes in transposition efficiency may be caused by the changed direction of *neo* gene transcription. For example, the machinery associated with the transcription of the *gfp* gene and the *neo* gene toward each other on pT7-*gfp*-*oen* may facilitate the formation of synaptic complexes, or alternatively the transcription of the *neo* gene in pT7-*gfp*-*neo* may cause run off transcripts through the right TIR to reduce its accessibility by transposases. Since high transposition efficiency is desirable for TE based genetic tools, the observation of a dramatic influence of the orientation of a cargo gene on transposition efficiency may facilitate the optimization of a TE based genetic tool.

In addition to demonstrating the use of MITE for genetic tools, these studies also provided a tool for genetic manipulation of yeast. Even though studies of yeast genes benefited from a number of efficient genetic tools, TEs can be very useful in mutagenesis. The endogeneous *Ty* retroelements have been shown to be efficient in mutagenesis [41]. Unlike retroelements, cut-and-paste TEs can excise from an insertion site therefore potentially leading to the restoration of the function of a mutated gene. Since the demonstration of transposition activity of the *Ac* element in yeast, transposition assay has been subsequently established in budding yeast for several other elements including the rice *Mariner*-like elements (*Osmar*5 and *Osmar*17) and *Stowaway* MITE *Ost*35, the housefly *Hermes* element and the flour beetle *TcBuster* element [14], [39], [43], [53], [54]. Insertion frequency was reported only for *Hermes* (2.5×10^−5^ per cell generation for the native transposase and 7×10^−2^ per cell generation for a hyperactive mutant transposase) and *TcBuster* (5×10^−4^ per cell generation). The transposition rate of the T7 derived constructs can be further increased upon improvement of the *Osmar*14 transposase, nevertheless, the insertion frequency of 1.12×10^−3^ per cell generation for T7-*neo* in our findings is within the range considered ideal for generating libraries with a large number of cells bearing a single insertion while minimizing double or multiple insertions [55]. Recently, a *piggyBac* based mutagenesis system has been demonstrated to be powerful in identifying both loss-of-function and gain-of-function alleles [56]. Excision of the *piggyBac* elements rarely leaves footprints, leading to complete reversion of the insertion sites. Since the transposition of *Mariner* elements and *Stowaway* MITEs generates TSDs of only two nucleotides, they are presumably less disruptive than *hAT* elements upon excision from a coding sequence.

## Supporting Information

Table S1 Excision assay raw data for Fig. 2A.
(DOCX)

Table S2 Excision assay raw data for Fig. 2D.
(DOCX)

Table S3 Insertions sites of hybrid Stowaway T7 derived vectors carrying cargo genes.
(DOCX)

Table S4 Insertions sites of L7 colonies.
(DOCX)

Table S5 Linked and unlinked transposition.
(DOCX)
