# Supplementary material for: A Rice Stowaway MITE for Gene Transfer in Yeast
Source: PLoS One. 2013 May 21;8(5):e64135. doi: 10.1371/journal.pone.0064135 (PMC3660474; doi:10.1371/journal.pone.0064135)
Supplement: Table S1 — Excision assay raw data for Fig. 2A. (DOCX) [file pone.0064135.s001.docx]

Supplemental Table 1. Excision assay raw data for Fig. 2A.

| **14 days after transformation** | | | | | | |
| --- | --- | --- | --- | --- | --- | --- |
|  | R1 | | R2 | | R3 | |
|  | YPD | -ADE | YPD | -ADE | YPD | -ADE |
| pT7+POsm14Tp | 230 | 1161 | 73 | 1057 | 89 | 958 |
| pT7+pRS413 | 55 | 0 | 40 | 0 | 58 | 0 |
| pT7Neo+POsm14Tp | 46 | 258 | 49 | 388 | 59 | 499 |
| pT7Neo+pRS413 | 41 | 0 | 51 | 0 | 105 | 0 |
| p14TIRNeo+POsm14Tp | 70 | 0 | 50 | 0 | 42 | 1 |
| p14TIRNeo+pRs413 | 38 | 0 | 47 | 0 | 39 | 0 |
| pOst35Neo+POsm14Tp | 88 | 0 | 108 | 0 | 54 | 1 |
| pOst35Neo+pRs413 | 66 | 0 | 62 | 0 | 48 | 0 |
| pT7GFP+POsm14Tp | 8 | 217 | 46 | 3890 | 3 | 185 |
| pT7GFP+pRS413 | 21 | 0 | 44 | 0 | 1 | 0 |
| pT7GFP-Neo+POsm14Tp | 122 | 98 | 123 | 583 | 100 | 58 |
| pT7GFP-Neo+pRS413 | 58 | 0 | 63 | 0 | 63 | 0 |

YPD, the number of colonies on YPD plates (dilution factor: 1.6 x 10^5^); -ADE, number of colonies on medium lacking adenine; R1,R2,R3: data obtained from replicate experiments using independent colonies on medium lacking histidine and uracil.
