# Supplementary material for: A Rice Stowaway MITE for Gene Transfer in Yeast
Source: PLoS One. 2013 May 21;8(5):e64135. doi: 10.1371/journal.pone.0064135 (PMC3660474; doi:10.1371/journal.pone.0064135)
Supplement: Table S2 — Excision assay raw data for Fig. 2D. (DOCX) [file pone.0064135.s002.docx]

Supplemental Table 2. Excision assay raw data for Fig. 2D.

| **9 days after transformation** | | | | | | |
| --- | --- | --- | --- | --- | --- | --- |
|  | R1 | | R2 | | R3 | |
|  | YPD | -ADE | YPD | -ADE | YPD | -ADE |
| pT7+PPOsm14TpTp | 83 | 160 | 58 | 210 | 35 | 303 |
| pT7+pRS413 | 40 | 0 | 38 | 0 | 29 | 0 |
| pT7Neo+POsm14Tp | 41 | 110 | 46 | 61 | 19 | 47 |
| pT7Neo+pRS413 | 30 | 0 | 24 | 0 | 14 | 0 |
| p14TIRNeo+POsm14Tp | 42 | 0 | 33 | 0 | 69 | 0 |
| p14TIRNeo+pRs413 | 27 | 0 | 38 | 0 | 128 | 0 |
| pOst35Neo+POsm14Tp | 47 | 1 | 48 | 0 | 57 | 0 |
| pOst35Neo+pRs413 | 41 | 0 | 35 | 0 | 39 | 0 |
| pT7GFP+POsm14Tp | 32 | 12 | 87 | 8 | 135 | 23 |
| pT7GFP+pRS413 | 51 | 0 | 36 | 0 | 21 | 0 |
| pT7GFP-Neo+POsm14Tp | 68 | 2 | 73 | 9 | 107 | 10 |
| pT7GFP-Neo+pRS413 | 29 | 0 | 50 | 0 | 13 | 0 |

YPD, the number of colonies on YPD plates (dilution factor: 1.6 x 10^5^); -ADE, number of colonies on medium lacking adenine; R1,R2,R3: data obtained from replicate experiments using independent colonies on medium lacking histidine and uracil.
