# Supplementary material for: A Rice Stowaway MITE for Gene Transfer in Yeast
Source: PLoS One. 2013 May 21;8(5):e64135. doi: 10.1371/journal.pone.0064135 (PMC3660474; doi:10.1371/journal.pone.0064135)
Supplement: Table S3 — Insertions sites of hybrid Stowaway T7 derived vectors carrying cargo genes. (DOCX) [file pone.0064135.s003.docx]

**Supplemental Table 3. Insertions sites of hybrid *Stowaway* T7 derived vectors carrying cargo genes**

| Construct | Molecule | Location (bp) | Annotation | Insertion Site Sequence |
| --- | --- | --- | --- | --- |
| T7-*neo* | 2μ | 711 | Intergenic | tctctagaaag...tataggaact |
|  |  | 714 | Intergenic | tctctagaaagta...taggaact |
|  |  | 800 | Intergenic | tgtaacgagcta...ctaaaatat |
|  |  | 2548 | RAF1, REP Antagonizing Factor or Recombinase Activating Factor. It increases 2 micron plasmid copy number by regulating the transcript levels of Rep1-Rep2. | tgtgatcta...agattctatctt |
|  |  | 2824 | RAF1, REP Antagonizing Factor or Recombinase Activating Factor. It increases 2 micron plasmid copy number by regulating the transcript levels of Rep1-Rep2. | taataatata...tagtctagcgc |
|  |  | 5248 | Intergenic | attcttgctta...ttacattata |
|  |  | 6248 | FLP, site-specific recombinase encoded on the 2-micron plasmid and required for 2-micron plasmid propagation. | cttgcttatta…cattataaaat |
|  | IV | 1191011 | GGA1, Golgi-localized protein that regulates Arf1p and Arf2p in a in order to facilitate traffic through the late Golgi. | ataaaaggta...tgaaggaagaa |
|  |  | 1276554 | RPB7, RNA polymerase II subunit B16 involved in transcribing RNA polymerase II complex by recruiting the 3'-end of processing factors and in the translation initiation process. | acagagcgta...tatgaagtatt |
|  |  | 1189271 | CNL1, protein of unknown function. | ccttgaaagta…tctgttattat |
|  | VII | 74545 | Intergenic | agtcacctata…tggtagttact |
|  | VIII | 381364 | Intergenic | tgctactta...attcttattttc |
|  | IX | 247160 | YIL058W, protein of unknown function. | gtgaaaggatt...gttgcttgttg |
|  | XIII | 505097 | Leucine tRNA | catattgctta…ctcaactgact |
|  | XVI | 794761 | Intergenic | gggctagcttc...tatgtatcaa |
| T7-*gfp* | III | 226552 | YCR061W, protein of unknown function. | acaaagatgta…catgaatagcgg |
|  | IV | 76850 | Intergenic | tttaacggata...cgtcttaata |
|  | V | 53333 | RPL12A, Ribosomal 60S subunit protein. | accaaaattcta...gactttgaa |
|  | XII | 705014 | YLR282C, protein of unknown function. | catagcttgta…ggttggtagca |
|  | XIII | 41996 | Intergenic | aataatacgta…ttcaatttatc |
|  | XV | 3961 | TY4 LTR, protein of unknown function. | taaataatac...tacaacttaac |
|  | XV | 762305 | ISU2, required for the synthesis of iron-sulfur proteins. | catactgcata…tatccgagtca |
| T7-*gfp*-*oen* | XII | 821325 | YLR345W, responsible for the metabolism of fructoso-2,6-bisphosphate. | aaagataaca...tacatggttat |
|  | XIII | 557422 | TIF34, eIF3i subunit of the core complex of translation initiation factor 3 (eIF3). | tatatatata...cgtgcgtatat |
|  | XV | 766716 | ARS1523, Autonomously Replicating Sequence. | ataatcatata...ctttctgccg |
