# Supplementary material for: A Rice Stowaway MITE for Gene Transfer in Yeast
Source: PLoS One. 2013 May 21;8(5):e64135. doi: 10.1371/journal.pone.0064135 (PMC3660474; doi:10.1371/journal.pone.0064135)
Supplement: Table S4 — Insertions sites of L7 colonies. (DOCX) [file pone.0064135.s004.docx]

**Supplemental Table 4. Insertions sites of L7 colonies**

| Colony # | Chromosome | Location | Annotation | Insertion Site Sequence | Occurrence |
| --- | --- | --- | --- | --- | --- |
| L7 line | 2 micron | 1786 | Rep1, regulates transcript levels of the FLP1 gene that promotes plasmid copy number. |  |  |
| 1 | XVI | 718811 | YPR092W, protein of unknown function. | ataataaacta...gtccgcaaca | 4 |
| 2 | XV | 81298 | Intergenic | aaataaagtgta...gtggcaga | 9 |
| 3 | XV | 81298 | Intergenic | aaataaagtgta...gtggcaga |  |
|  | XIII | 793635 | Intergenic | gacaattgatta...cgtagggaa | 3 |
| 4 | XVI | 718811 | YPR092W, protein of unknown function. | ataataaacta...gtccgcaaca |  |
| 6 | IX | 15455 | VTH1, putative membrane glycoprotein. | ggaacccgact...taattgaata | 2 |
|  | XVI | 718811 | YPR092W, protein of unknown function. | ataataaacta...gtccgcaaca |  |
| 8 | XV | 81298 | Intergenic | aaataaagtgta...gtggcaga |  |
| 9 | XV | 81298 | Intergenic | aaataaagtgta...gtggcaga |  |
|  | XIII | 793635 | YMR262W, Protein of unknown function. | gacaattgatta...cgtagggaa |  |
| 11 | XV | 81239 | Intergenic | aatttttcttta...aatatacaa | 1 |
|  | XV | 81298 | Intergenic | aaataaagtgta...gtggcaga |  |
| 12 | XV | 81298 | Intergenic | aaataaagtgta...gtggcaga |  |
|  | IX | 187177 | Intergenic | ttttttcaattt...taatgtaca | 1 |
| 13 | XII | 460728 | 5S rDNA, non-transcribed region of the rDNA repeat and required for rDNA repeat expansion. | aatagtaact...tacatacatta | 2 |
| 14 | IX | 15455 | VTH1, putative membrane glycoprotein. | ggaacccgact...taattgaata |  |
|  | XV | 81298 | Intergenic | aaataaagtgta...gtggcaga |  |
| 15 | IV | 133192 | LYS20, Homocitrate synthase isozyme. | attagagcaa...taggcaataat | 1 |
|  | IX | 187665 | LYS12, Homo-isocitrate dehydrogenase, which is required biosynthesis of lysine. | tagttatata...aatatgtcgca | 2 |
| 16 | IX | 187665 | LYS12, Homo-isocitrate dehydrogenase, which is required biosynthesis of lysine. | tagttatata...aatatgtcgca |  |
| 17 | IX | 16143 | ARS904, Autonomously Replicating Sequence. | gacaagtagg...taggtcttttt | 1 |
| 18 | XII | 460728 | 35S rDNA, non-transcribed region of the rDNA repeat and required for rDNA repeat expansion. | aatagtaact...tacatacatta |  |
|  | XV | 81298 | Intergenic | aaataaagtgta...gtggcaga |  |
| 20 | XV | 81298 | Intergenic | aaataaagtgta...gtggcaga |  |
|  | XVI | 718811 | YPR092W, protein of unknown function. | ataataaacta...gtccgcaaca |  |
|  | XIV | 202026 | LAP3, Cysteine aminopeptidase with homocysteine-thiolactonase activity. | gataacgtgta...tattattatt | 1 |
| 22 | X | 15823 | VTH1, putative membrane glycoprotein. | ataataatata...cagcgcaactt | 1 |
| 24 | XVI | 718809 | SNR70, predicted to guide 2'-O-methylation of small subunit (SSU) rRNA at position C1639. | gttgcggacta…gtttattatga | 1 |
| 25 | V | 353714 | Intergenic | attataatgta...agcgtttgttt | 1 |
|  | XIII | 793635 | YMR262W, protein of unknown function. | gacaattgatta...cgtagggaa |  |
