# Supplementary material for: A Rice Stowaway MITE for Gene Transfer in Yeast
Source: PLoS One. 2013 May 21;8(5):e64135. doi: 10.1371/journal.pone.0064135 (PMC3660474; doi:10.1371/journal.pone.0064135)
Supplement: Table S5 — Linked and unlinked transposition. (DOCX) [file pone.0064135.s005.docx]

**Supplemental Table 5. Linked and unlinked transposition**

| **Colony on transposase induction medium** | **GFP expression of  *ade*2 revertants on galactose medium** | | **GFP expression of colonies on 5-FOA galactose medium** | |
| --- | --- | --- | --- | --- |
| 1 | Green | 54 | Green | 12 |
|  |  |  | Not green | 8 |
|  | Not green | 12 |  | |
| 2 | Green | 61 | Green | 6 |
|  |  |  | Not green | 14 |
|  | Not green | 8 |  | |
| 3 | Green | 42 | Green | 13 |
|  |  |  | Not green | 7 |
|  | Not green | 5 |  | |
| 4 | Green | 41 | Green | 9 |
|  |  |  | Not green | 11 |
|  | Not green | 5 |  | |

Each 5-FOA galactose plate had hundreds of colonies, either all of them express GFP or none of them express GFP (Fig 6).
